# Supplementary material for: Identification and functional analysis of non-coding regulatory small RNA FenSr3 in Bacillus amyloliquefaciens LPB-18
Source: PeerJ. 2023 May 15;11:e15236. doi: 10.7717/peerj.15236 (PMC10194069; doi:10.7717/peerj.15236)
Supplement: Supplemental Information 4 [file peerj-11-15236-s004.zip › KO/CK-vs-T1_map/map01501.html]

KEGG PATHWAY: beta-Lactam resistance - Reference pathway


|  |  |
| --- | --- |
| **beta-Lactam resistance - Reference pathway** |  |

[
Pathway menu
| Organism menu
| Pathway entry
| Show description
| User data mapping
]

The beta-lactam antibiotics are the most widely used group of antibiotics, which exert their effect by interfering with the structural crosslinking of peptidoglycans in bacterial cell walls. Over the past decades, a rapid increase in the number of beta-lactam-resistant clinical isolates is observed. Bacterial resistance to beta-lactam antibiotics can be achieved by any of the following strategies: producing inactivating enzymes called beta-lactamases, altering the beta-lactam targets of penicillin-binding proteins (PBPs), reducing transport of beta-lactams into the periplasmic space via changes in porins, and using the mechanisms for exclusion of beta-lactams by way of efflux pumps. Resistance may result from intrinsic properties of organisms, through gene mutations, and through plasmid- and transposon-specified genes.

|  |  |  |
| --- | --- | --- |
| Reference pathway | 184% 150% 122% 100% 82% 67% 55% | 图片下载 |
